# Operationalizing ASSIST-based indicators to estimate the prevalence and correlates of problematic cannabis involvement among U.S. college students

**DOI:** 10.1186/s42238-026-00446-4

**Published:** 2026-05-14

**Authors:** Fares Qeadan, Ashlie McCunn, Anumitha Aravindan, Benjamin Tingey

**Affiliations:** Parkinson School of Health Sciences and Public Health, Loyola University Chicago, 2160 S 1st Ave, Maywood, IL 60153 USA

**Keywords:** problematic cannabis involvement, cannabis involvement severity, ASSIST, college students, depression, anxiety, mental health

## Abstract

**Background:**

Cannabis use and cannabis-related harms remain important public health concerns among college-age adults. However, nationally representative estimates that characterize the severity of problematic cannabis use in U.S. college students are limited. Using pooled American College Health Association-National College Health Assessment (ACHA-NCHA) III data, we developed a screening-based index of cannabis involvement severity and examined mental health and other correlates.

**Methods:**

This cross-sectional study used pooled ACHA-NCHA III data from Fall 2019 to Spring 2024. Cannabis involvement severity was operationalized using cannabis-specific items from the Alcohol, Smoking and Substance Involvement Screening Test (ASSIST) module embedded in the ACHA-NCHA III (e.g., use frequency, craving/urge, difficulty controlling use, role impairment, and cannabis-related problems). Indicator responses were summed and categorized as no/low involvement (0–1), mild (2–3), moderate (4–5), or severe/high involvement (≥ 6). We estimated prevalence overall and among students with lifetime cannabis use. Ordinal logistic regression identified correlates of greater involvement severity among those with lifetime cannabis use. Modified Poisson and log-linear models assessed associations between involvement severity and mental health indicators among students with cannabis use history.

**Results:**

The prevalence of elevated problematic cannabis involvement (score ≥ 2) was 8.66% among all students and 20.77% among those with lifetime cannabis use; among students with cannabis use history, 7.32% had mild involvement, 6.88% moderate involvement, and 6.56% severe/high involvement. Greater involvement severity was more common among males, American Indian/Alaskan Native/Non-Hispanic (NH) Black/NH Other/NH multiracial students (compared with NH White), undergraduates (compared with Master’s/PhD students), and those not living on campus, and was associated with greater co-occurring tobacco/other substance use and greater counts of chronic mental health conditions. Compared to students with no/low cannabis involvement, those with mild/moderate/severe involvement had higher prevalence of anxiety, depression, other mental health conditions, and mental health service receipt, as well as higher non-specific serious mental illness, loneliness, and suicide behavior scores.

**Conclusions:**

Problematic cannabis involvement is common among U.S. college students and shows a graded association with adverse mental health indicators. These findings support targeted screening and integrated behavioral health services for students reporting more severe patterns of cannabis involvement.

**Supplementary Information:**

The online version contains supplementary material available at 10.1186/s42238-026-00446-4.

## Introduction

Due to increasing accessibility and efforts towards decriminalization in the United States (U.S.), cannabis has risen in popularity as one of the most commonly used recreational drugs (Cerdá et al. 2020). While many individuals use cannabis without meeting criteria for a disorder, cannabis-related harms are concentrated among higher frequency use patterns (Rock et al. 2022). Recent research shows that individuals who use cannabis have about a 30% chance of developing Cannabis Use Disorder (CUD); a problematic pattern of cannabis use leading to clinically significant impairment or distress (Hasin et al. 2015). Frequent cannabis use and CUD are associated with multitude of adverse health outcomes including lung cancer (Callaghan et al. 2013), cardiovascular issues (Shah et al. 2021), cognitive impairment (Kroon et al. 2020), as well as mental health conditions including psychosis and suicidality (Ahrens et al. 2025; Han et al. 2021). Current estimates show college-age populations engage in cannabis use at higher rates than other age groups. The 2023 Monitoring the Future Survey found over 40% of young adults (19–30 years old) reported cannabis use in the past year, 28.7% reported use in the past month, and 10.4% reported daily cannabis use (Patrick et al. 2024). Results from the 2024 National Survey on Drug Use and Health (NSDUH) indicate CUD prevalence is highest among 18–25-year-olds (15.8%) compared to adolescents (4.7%) and adults 26 years or older (6.1%) (Substance Abuse and Mental Health Services Administration, 2025). Several studies among young adults have found cannabis use is associated with reduced income, greater debt, and lower life satisfaction (Tartaglia et al. 2017; Thompson et al. 2019). College students that report frequent cannabis use have also exhibited significantly lower grade point averages, were less likely to be enrolled senior year, and less likely to plan to graduate on time compared to students who did not use cannabis (Suerken et al. 2016).

While previous research has identified risk factors for CUD development (Ferland and Hurd 2020; Hinckley et al. 2024), very few studies have investigated the specific predictors of CUD *severity* especially among a high-risk group of college-age adults. One longitudinal study involving a cohort of young Swiss men found that predictors associated with persistently severe CUD included major depression severity, attention deficit hyperactivity disorder (ADHD) severity, antisocial personality disorder severity, number of friends using substances, and personality traits including neuroticism-anxiety and sociability (Marmet et al. 2021). Another study using responses from young adults and adolescents from the United Kingdom (UK) found that adolescent age (16–17 years) compared to young adults (26–29 years) and having CUD at baseline (compared to no CUD) predicted more severe CUD at follow-up (Skumlien et al. 2025). Additionally, as high-potency cannabis products have become more widely popular in recent years, emerging research has identified these products, such as cannabis concentrates, as a particular risk factor for problematic cannabis use, dependence, and mental health conditions (Bidwell et al. 2021; Hines et al. 2020; Petrilli et al. 2022). Results from this study could augment the current understanding of CUD development by identifying various CUD severity predictors among a comprehensive sample of U.S. young adults.

Mental health conditions have a complex relationship with development of cannabis dependence and CUD (Ferland and Hurd 2020). Some research has shown that baseline mood, anxiety, or substance use conditions do predict future cannabis use and CUD (Wittchen et al. 2007). While other studies suggest that frequent cannabis use and dependence are associated with increased risk of substance use disorders (Blanco et al. 2016), psychotic symptoms (West and Sharif 2023), and depression and anxiety (Hammond et al. 2020). When investigating this relationship among youth, a study using data from NSDUH found that adolescents with CUD had substantially higher odds of major depressive episode (odds ratio = 2.42) and suicidal ideation (odds ratio = 2.92) compared with adolescents with no past-year cannabis use (Sultan et al. 2023). Another study on adolescents in Norway found that an increase from no cannabis use to use greater than 10 times per year was associated with an increased risk for anxiety and suicidal ideation (Gripe et al. 2024). To our knowledge, no studies have estimated how mental health indicators associate with the severity of CUD, especially among college-age adults. It is crucial to better understand both predictors and outcomes associated with CUD severity to determine if early intervention can be applied to individuals exhibiting problematic patterns of use among a high-risk population of young adults.

Given the range of harms associated with heavier or more problematic cannabis use, including academic disruption, injury risk, and co-occurring mental health burden, there is a need for scalable surveillance approaches that go beyond simple use prevalence. Existing estimates of problematic cannabis use in college populations vary widely across studies due to differences in instruments, thresholds, and sampling frames, and many prior analyses rely on smaller or geographically limited cohorts. Leveraging the American College Health Association-National College Health Assessment (ACHA-NCHA) III provides an opportunity to quantify problematic cannabis involvement severity and its correlates in a large, contemporary national (multi-institution) sample of U.S. college students (not necessarily nationally representative), informing campus prevention and early intervention efforts.

Embedded as a module within the ACHA-NCHA III survey is The Alcohol, Smoking, and Substance Involvement Screening Test (ASSIST). The ASSIST was developed by the World Health Organization as a brief, standardized screening tool to characterize substance involvement severity and support triage and brief intervention in clinical and public health settings (Humeniuk et al. 2010). The ASSIST has demonstrated acceptable psychometric properties with established concurrent validity, construct validity, and discriminative validity against other standardized screening, diagnostic, and severity assessment tools for substance use and mental health (Humeniuk et al. 2008). While ASSIST provides a substance specific involvement score (SSIS; ranging from 0 to 39) for all substances, including cannabis, the SSIS itself is designed as a rapid screening tool, that was originally validated against version IV of the Diagnostic and Statistical Manual of Mental Disorders (DSM) (Humeniuk et al. 2008). Moderate risk scores (SSIS 11–26 for alcohol, 4–26 for all other substances) recommend brief intervention, and high risk scores (SSIS ≥ 27) recommend more intensive intervention (Humeniuk et al. 2010). When analyzed for research purposes, these moderate and high risk cutoffs may, respectively, overcapture and undercapture general estimates more aligned with actual problematic patterns of cannabis use such as CUD. Furthermore, few studies have utilized the current version of DSM (5th edition) in their ASSIST-based studies and are also limited to restricted populations with overly sensitive risk cutoffs not applicable to college student populations. Additionally, to our understanding, existing studies have not incorporated substance use severity in their aims. Given these limitations, further work is required to construct a screening-based index of problematic cannabis involvement severity that utilizes the current DSM-5 criteria, to facilitate more precise and reliable analytical estimates of problematic use in college populations.

In addition to limited generalizability of existing estimates and the need to better understand cannabis-related harms in college populations, few studies have characterized the severity of problematic cannabis use at the national level using campus surveillance data. Moreover, the association between gradations of problematic cannabis involvement and mental health conditions among college-age adults remains underexplored. This study aims to develop a screening-based index of problematic cannabis involvement using ACHA-NCHA III cannabis-specific ASSIST items to estimate the prevalence of elevated involvement in students overall and among students with cannabis use history. Further among those with cannabis use history, this study seeks to identify correlates of greater involvement severity and quantify mental health indicators across involvement severity levels.

## Materials and methods

### Data source, sample, and design

Data for this study were obtained from the ACHA-NCHA survey constructed for Fall 2019 to Spring 2024 semesters (version III), referred to as the ACHA-NCHA III (Lederer and Hoban 2022). The ACHA-NCHA III is administered to students by participating colleges and universities across the U.S. and provides access to a large national cohort of students. It asks about a wide range of health behaviors and attitudes, including alcohol, tobacco, and other drug use, sexual health, mental health, physical health, personal safety and violence, food insecurity and homelessness, demographic characteristics, and institutional/campus characteristics. The ACHA-NCHA III provides broad national coverage across participating institutions but is not intended to produce nationally representative estimates of all U.S. college students. This study aggregated data from Fall 2019 to Spring 2024 and thus followed a cross-sectional design. Two groups were assessed including all students and students reporting any lifetime use of cannabis by answering in the affirmative to the question: “In your life, which of the following substances have you ever used: Cannabis (marijuana, weed, hash, edibles, vaped cannabis, etc.) *[Please report nonmedical use only.]”* Among these groups, student respondents were further restricted to those with male or female sex assigned at birth. This study was deemed exempt by the Loyola University Chicago Institutional Review Board (LU# 219802) under 45 CFR 46.104(d)(4) as secondary research using de-identified data.

### Outcomes

Primary outcomes included a screening-based measure of problematic cannabis involvement and its severity level. Because the ACHA-NCHA III does not include a formal DSM-5 cannabis use disorder diagnosis, we operationalized cannabis involvement severity using cannabis-specific items from the ASSIST module embedded in the survey, capturing domains such as use frequency, craving/urge, difficulty controlling use, role impairment, and cannabis-related problems. Domains mostly comprised occurrences over the past 3 months. Supplemental Table 1 lists the included cannabis involvement domains, ASSIST-specific item(s) utilized to approximate the domain, and scoring rules. Items were measured on ordinal scales, for example: “In the past 3 months, how often have you used [cannabis]?” with responses ranging from “never” to “daily or almost daily.” The 11 domains were dichotomized into binary indicators (1 = endorsed, 0 = not endorsed) based on whether responses to the relevant items reflected a strong, or consistent, affirmative presence of each domain. Indicators, ranging from 0 to 11, were summed and categorized as no/low involvement (0–1), mild (2–3), moderate (4–5), and severe/high involvement (≥ 6) to mirror symptom categorization of the DSM-5 (Hasin Deborah et al. 2013). Scores ≥ 2 represent elevated problematic cannabis involvement; an operational/heuristic threshold. Participants were required to have responses to at least 8 of the 11 indicators (~ 73% available responses) to minimize bias from missingness; 1.11% of respondents were removed.

Secondary outcomes comprised mental health indicators and included binary responses to lifetime diagnosis history of anxiety, depression, and other mental health conditions (attention deficit hyperactivity disorder, bipolar disorder, borderline personality disorder, obsessive compulsive disorder, post-traumatic stress disorder, schizophrenia, eating disorder, or other mental health/psychological disorder) (Hinckley et al. 2023; Lowe et al. 2019). Additionally, lifetime binary receipt of psychological or mental health services was also assessed. Continuous outcomes of mental health well-being, measured by validated constructs, included the Kessler 6 non-specific serious mental illness score (range: 0–24) (Kessler et al. 2002), the UCLA three-item loneliness scale (range: 3–9) (Hughes et al. 2004), the suicide behavior questionnaire-revised screening score (3–18) (Osman et al. 2001), the Diener flourishing scale (8–56) (Diener et al. 2010), and the Connor-Davidson resilience scale (0–8) (Connor and Davidson 2003; Vaishnavi et al. 2007), with higher scores indicative of higher presence of particular outcomes.

### Exposures and additional measures

Exposures of interest included sociodemographic factors of age, sex, race/ethnicity, academic year, housing status, and census region. For mental health indicators, cannabis involvement severity level served as the exposure of interest. Additional measures, including those identified from literature as notable risk factors for problematic cannabis involvement and problematic cannabis involvement severity, included relationship status, campus enrollment size, semester/year, parent educational attainment, working status, challenges with academics, food insecurity, chronic physical/mental conditions, recent tobacco/alcohol/other substance use (in the last three months), recent stress levels (in the last 30 days), positive suicide screening, positive loneliness screening, and campus climate (Arria et al. 2013; Bidwell et al. 2014; Caldeira et al. 2008; Hasin et al. 2016; Leung et al. 2020; Marmet et al. 2021; Phillips et al. 2018; Pitman et al. 2024; Seidel et al. 2020; Skumlien et al. 2025).

### Statistical analysis

Characteristics were presented for all students and by cannabis use history. The prevalence of elevated problematic cannabis involvement (score ≥ 2) was tabularized and visualized over time, overall and stratified by key sociodemographic characteristics. Cannabis involvement severity levels were also tabularized. To assess associations between student characteristics and cannabis involvement severity among those with lifetime cannabis use, ordinal logistic regression was used to estimate adjusted odds ratios (aORs) and 95% confidence intervals (CIs). Forest plots visualized these associations. To assess associations between cannabis involvement severity and mental health indicators among those with lifetime cannabis use, modified Poisson regression was used for binary outcomes to estimate adjusted prevalence ratios (aPRs), and log-linear regression was used for continuous construct outcomes to estimate exponentiated adjusted coefficients ($\:{e}^{{\widehat{\beta\:}}_{ADJ}}$) representing percentage changes by severity level. All hypothesis tests were two-sided with a significance level of 5%. SAS version 9.4 (SAS Institute, INC) was used for all analyses.

### Supplemental validation analysis

We conducted an exploratory factor analysis of the cannabis involvement indicators to evaluate dimensionality and support the use of an overall involvement severity score. Specifically, an iterated principal factor extraction with oblique promax (power = 3) rotation was conducted with 4 factors extracted. To assess the comparability of our screening-based cannabis involvement construct to an ACHA-NCHA III question indicating clinician-diagnosed alcohol or other drug-related abuse or addiction (“Have you ever been diagnosed by a healthcare or mental health professional with an alcohol or other drug-related abuse or addiction?”), correlations between the binary SUD indicator and elevated problematic cannabis involvement (score ≥ 2) were calculated in the overall student sample via the phi-coefficient (*r*_*ϕ*_) and tetrachoric correlation (*r*_*tet*_). We additionally assessed the comparability of our cannabis involvement severity construct to the ASSIST cannabis SSIS, among students with lifetime cannabis use, treating both constructs on their original ordinal scales, with Spearman’s rank-order correlation (*r*_*s*_). We also compared the prevalence of elevated problematic cannabis involvement to the prevalence of moderate/high cannabis SSIS (SSIS ≥ 4) and high cannabis SSIS (SSIS ≥ 27) among those with lifetime cannabis use. We then fitted separate regression models for SUD, moderate/high cannabis SSIS (SSIS ≥ 4), and elevated problematic cannabis involvement to compare predictor patterns. Lastly, modified Poisson regression assessed associations between SUD/moderate or high cannabis SSIS/elevated problematic cannabis involvement and binary mental health indicators in the overall student group.

## Results

### Descriptive statistics

The total number of student respondents included in analysis was 534,816. Of these, 41% (219,097) had lifetime cannabis use and 59% (315,719) never used cannabis (Table 1). Overall students had a median age of 21 years, nearly 70% were female, over half were Non-Hispanic (NH) White, 15.4% were Hispanic, and 14.6% were NH Asian or Pacific Islander (API). Over half of students were not in a relationship, over three-fourths were undergraduates, and over 44% lived off campus. Under half of respondents had one or more chronic physical conditions, and over half of respondents had one or more chronic mental health conditions. While only ~ 20% of the sample had recent tobacco use, over 65% had recent alcohol use. There were 29% of students that reported high recent stress levels and 49% that reported moderate stress (Table 1). When comparing characteristics between cannabis use history, all characteristics demonstrated significant differences. Notably, those with lifetime cannabis use were more likely to be NH White, NH Multiracial (chi-squared test-statistic [χ^2^]: 15,961.96, *p* < 0.001), in a relationship (χ^2^: 6,018.85, *p* < 0.001), live off-campus (χ^2^: 11,961.07, *p* < 0.001), have food insecurity (χ^2^: 1,875.12, *p* < 0.001), have more chronic physical/mental health conditions (chronic physical conditions χ^2^: 5,549.20, *p* < 0.001; chronic mental health conditions χ^2^: 32,237.76, *p* < 0.001), have recently used tobacco/alcohol/other substances (tobacco χ^2^: 77,695.74, *p* < 0.001; alcohol χ^2^: 103,534.00, *p* < 0.001; other substance χ^2^: 50,873.72, *p* < 0.001), have higher recent stress levels (χ^2^: 4,519.99, *p* < 0.001), test higher on suicide and loneliness screenings (suicide Wilcoxon rank-sum test statistic [Z]: 157.74, *p* < 0.001; loneliness *Z*: 29.40, *p* < 0.001), and were less likely to report positive campus climate (χ^2^: 1,004.55, *p* < 0.001), live on-campus or with family (χ^2^: 11,961.07, *p* < 0.001), and be NH API (χ^2^: 15,961.96, *p* < 0.001) than students who reported no cannabis use (Table 1).

**Table 1:** Characteristics of student participants (stratified by cannabis use history^1^) among those with available responses for cannabis involvement construction

Characteristic	All students	Lifetime cannabis use	No cannabis use	$\:{\boldsymbol{x}}_{4}^{2}$
	*n* (%^2^)	*n* (%^2^)	*n* (%^2^)	
Overall	534,816	219,097 (41.00^3^)	315,719 (59.00^3^)	
Age^5^	21.00 (19.00–24.00)	21. 00 (20. 00–25.00)	21.00 (19.00–24.00)	75.65^***6^
Sex				449.19^***^
Female	367,652 (69.05)	154,328 (70.66)	213,324 (67.93)	
Male	164,791 (30.95)	64,081 (29.34)	100,710 (32.07)	
Race/ethnicity				15,961.96^***^
AI/AN^7^	11,633 (2.18)	5291 (2.41)	6342 (2.01)	
Hispanic	82,343 (15.40)	33,678 (15.37)	48,665 (15.41)	
NH API^8^	77,891 (14.56)	17,863 (8.15)	60,028 (19.01)	
NH Black	26,281 (4.91)	8876 (4.05)	17,405 (5.51)	
NH White	293,102 (54.80)	135,518 (61.85)	157,584 (49.91)	
NH Other	14,687 (2.75)	4169 (1.90)	10,518 (3.33)	
NH Multiracial	28,879 (5.40)	13,702 (6.25)	15,177 (4.81)	
Relationship status				6,018.85^***^
Not in a relationship	274,789 (51.66)	99,598 (45.61)	175,191 (55.86)	
Relationship, not partnered/married	202,983 (38.16)	96,231 (44.07)	106,752 (34.04)	
Married/partnered	54,192 (10.19)	22,527 (10.32)	31,665 (10.10)	
Academic year				256.46^***^
Undergraduate	401,211 (75.34)	162,124 (74.22)	239,087 (76.12)	
Master’s or PhD	124,083 (23.30)	53,133 (24.32)	70,950 (22.59)	
Other/not seeking degree	7242 (1.36)	3193 (01.46)	4049 (1.29)	
Housing status				11,961.07^***^
Fraternity	6867 (1.29)	3459 (01.58)	3408 (1.09)	
On-campus	180,132 (33.84)	64,676 (29.61)	115,456 (36.78)	
Family	100,600 (18.90)	31,592 (14.46)	69,008 (21.99)	
Off-campus	236,067 (44.35)	115,116 (52.70)	120,951 (38.53)	
Temp/unhoused	2542 (0.48)	1231 (00.56)	1311 (0.42)	
Other	6119 (1.15)	2378 (01.09)	3741 (1.19)	
Region				2,600.09^***^
NE	113,333 (21.19)	49,448 (22.57)	63,885 (20.23)	
MW	121,635 (22.74)	47,959 (21.89)	73,676 (23.34)	
S	149,987 (28.04)	54,656 (24.95)	95,331 (30.19)	
W	149,861 (28.02)	67,034 (30.60)	82,827 (26.23)	
Total enrollment				72.26^***^
<2,500 students	39,639 (7.41)	16,385 (07.48)	23,254 (7.37)	
2,500-4,999 students	41,925 (7.84)	17,736 (08.10)	24,189 (7.66)	
5,000–9,999 students	84,411 (15.78)	33,705 (15.38)	50,706 (16.06)	
10,000–19,999 students	107,827 (20.16)	44,369 (20.25)	63,458 (20.10)	
≥20,000 students	261,014 (48.80)	106,902 (48.79)	154,112 (48.81)	
Semester				1,991.36^***^
Fall 2019	38,386 (7.18)	14,537 (06.63)	23,849 (7.55)	
Spring 2020	49,872 (9.33)	21,561 (09.84)	28,311 (8.97)	
Fall 2020	13,230 (2.47)	4764 (02.17)	8466 (2.68)	
Spring 2021	95,498 (17.86)	40,778 (18.61)	54,720 (17.33)	
Fall 2021	32,128 (6.01)	11,771 (05.37)	20,357 (6.45)	
Spring 2022	68,460 (12.80)	28,650 (13.08)	39,810 (12.61)	
Fall 2022	33,432 (6.25)	12,683 (05.79)	20,749 (6.57)	
Spring 2023	77,227 (14.44)	34,689 (15.83)	42,538 (13.47)	
Fall 2023	24,144 (4.51)	8053 (03.68)	16,091 (5.10)	
Spring 2024	102,439 (19.15)	41,611 (18.99)	60,828 (19.27)	
Parent educational attainment				2,012.00^***^
Did not finish high school	23,665 (4.51)	7290 (03.36)	16,375 (5.31)	
High school or GED	76,758 (14.62)	28,933 (13.36)	47,825 (15.52)	
Some college	42,650 (8.13)	18,974 (08.76)	23,676 (07.68)	
Associates/Bachelor’s	198,224 (37.76)	81,703 (37.71)	116,521 (37.80)	
Master’s/PhD	183,592 (34.98)	79,745 (36.81)	103,847 (33.69)	
Working				2,882.83^***^
No	209,505 (39.60)	76,529 (35.27)	132,976 (42.61)	
Yes	319,541 (60.40)	140,446 (64.73)	179,095 (57.39)	
Problems or challenges with academics				267.65^***^
No	275,901 (51.85)	110,234 (50.50)	165,667 (52.78)	
Yes	256,260 (48.15)	108,044 (49.50)	148,216 (47.22)	
Food insecurity				1,875.12^***^
No	432,126 (81.82)	171,398 (79.06)	260,728 (83.73)	
Yes	96,046 (18.18)	45,394 (20.94)	50,652 (16.27)	
Chronic physical conditions				5,549.20^***^
0	276,742 (52.12)	101,052 (46.35)	175,690 (56.14)	
1	132,103 (24.88)	57,987 (26.60)	74,116 (23.68)	
2	63,678 (11.99)	29,952 (13.74)	33,726 (10.78)	
3	29,082 (5.48)	14,318 (06.57)	14,764 (4.72)	
≥4	29,355 (5.53)	14,726 (06.75)	14,629 (4.67)	
Chronic mental health conditions				32,237.76^***^
0	231,165 (43.34)	66,356 (30.34)	164,809 (52.37)	
1	109,094 (20.45)	46,698 (21.35)	62,396 (19.83)	
2	59,687 (11.19)	28,267 (12.92)	31,420 (9.98)	
3	62,951 (11.80)	34,108 (15.60)	28,843 (9.17)	
≥4	70,487 (13.22)	43,279 (19.79)	27,208 (8.65)	
Recent^9^ tobacco use				77,695.74^***^
No	426,540 (79.84)	134,542 (61.48)	291,998 (92.59)	
Yes	107,684 (20.16)	84,307 (38.52)	23,377 (7.41)	
Recent^9^ alcohol use				103,534.00^***^
No	182,662 (34.28)	20,069 (09.18)	162,593 (51.72)	
Yes	350,227 (65.72)	198,449 (90.82)	151,778 (48.28)	
Recent^9^ other substance use				50,873.72^***^
No	293,437 (54.87)	87,149 (39.78)	206,288 (65.34)	
Yes	42,974 (8.04)	35,451 (16.18)	7523 (2.38)	
Unknown	198,405 (37.10)	96,497 (44.04)	101,908 (32.28)	
Recent stress levels				4,519.99^***^
No stress	8364 (1.57)	1829 (00.84)	6535 (2.08)	
Low	109,373 (20.52)	38,608 (17.67)	70,765 (22.50)	
Moderate	262,458 (49.24)	107,312 (49.11)	155,146 (49.34)	
High	152,790 (28.67)	70,763 (32.38)	82,027 (26.08)	
Suicide screening ^5,10^	4.00 (3.00–7.00)	5.00 (3.00–8.00)	3.0 (3.00–6.00)	157.74^***6^
Loneliness screening ^5,11^	6.00 (4.00–7. 00)	6.00 (4.00–7.00)	5.0 (4.00–7.00)	29.40^***6^
Positive campus climate				1,004.55^***^
No	211,638 (39.71)	92,305 (42.26)	119,333 (37.94)	
Yes	321,343 (60.29)	126,130 (57.74)	195,213 (62.06)	

1 cannabis use history defined by students answering in the affirmative to the question: “In your life, which of the following substances have you ever used: Cannabis (marijuana, weed, hash, edibles, vaped cannabis, etc.) [Please report nonmedical use only.]” 2 column % (unless otherwise noted) 3 row % (out of total *n* = 534,816) 4 Chi-squared test statistic (unless otherwise noted), *p < 0.05, *p < 0.01, ***p < 0.001 5 median (interquartile range) 6 Wilcoxon rank-sum test statistic, *p < 0.05, *p < 0.01, ***p < 0.001 7 American Indian or Alaskan Native 8 Asian or other pacific islander 9 usage within the past 3 months 10 Suicide Behavior Questionnaire-Revised (SBQR) Screening 11 UCLA Loneliness Scale Score

**Fig. 1:**
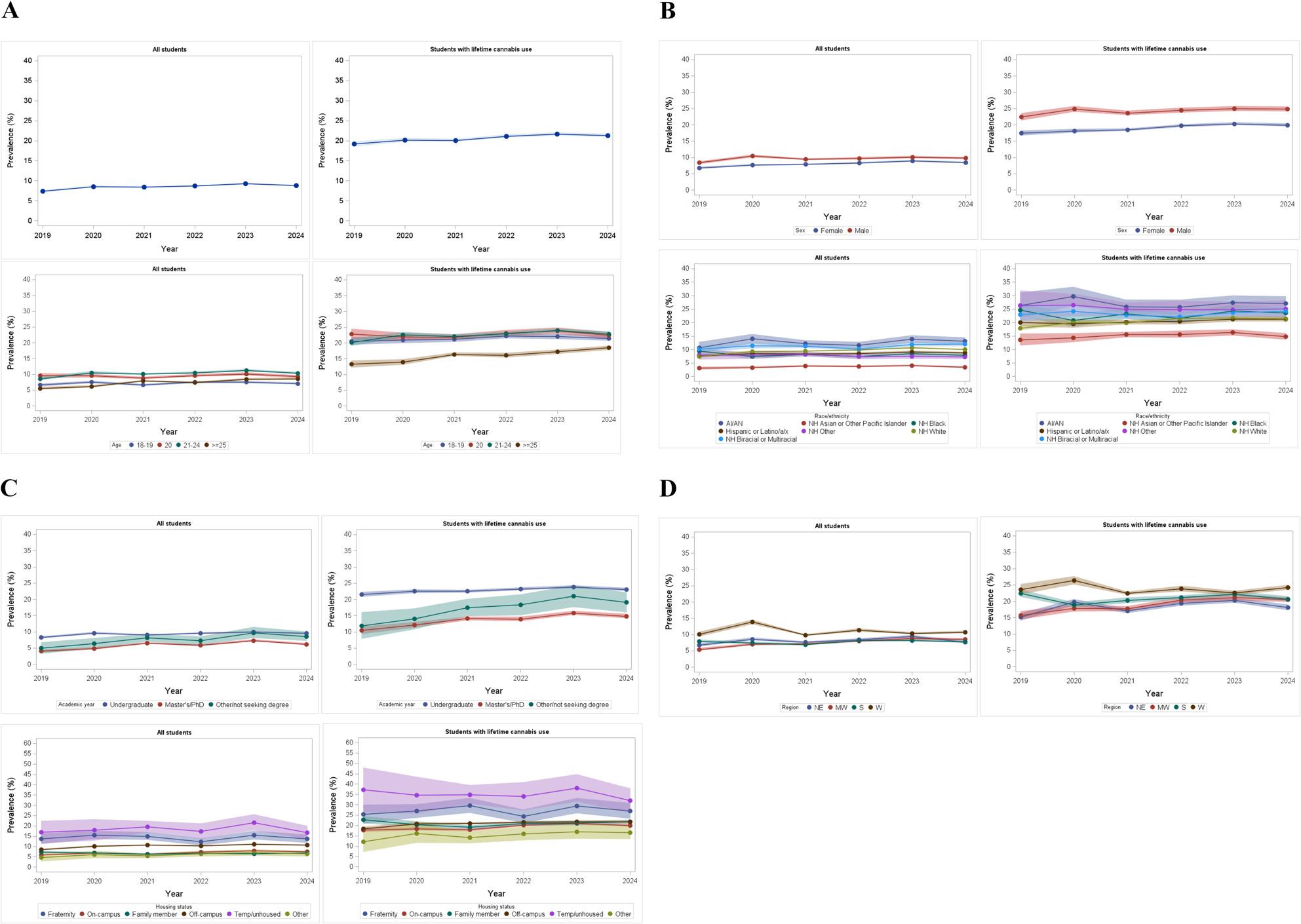
**A**. Prevalence (95% CI) of elevated problematic cannabis involvement (score ≥2) over time (among all students and those with lifetime cannabis use) overall and by age. **B**. Prevalence (95% CI) of elevated problematic cannabis involvement (score ≥2) over time (among all students and those with lifetime cannabis use) by sex and race/ethnicity. **C**. Prevalence (95% CI) of elevated problematic cannabis involvement (score ≥2) over time (among all students and those with lifetime cannabis use) by academic year and housing status. **D**. Prevalence (95% CI) of elevated problematic cannabis involvement (score ≥2) over time (among all students and those with lifetime cannabis use) by region

Prevalence of elevated problematic cannabis involvement (score ≥ 2) among all students and students with lifetime cannabis use is displayed in Table 2 along with trends over time in Fig. 1A-D. Among all students, the prevalence (%) was 8.66 (95% CI: 8.58, 9.73). Among those with lifetime cannabis use, the prevalence was 20.77 (95% CI: 20.60, 20.94). Youngest (18–19 years % [95% CI]: 7.13 [7.00, 7.26]) and oldest (≥ 25 years: 7.72 [7.57, 7.87]) age groups demonstrated the lowest prevalence among all students. Among those with lifetime cannabis use, the oldest students again had the lowest prevalence (16.46 [16.16, 16.76]), but the youngest students had similarly high prevalence as other age groups. Males had higher prevalence than females in both cohorts (males among all students: 9.69 [9.55, 9.84], females among all students: 8.18 [8.10, 8.27]; males with cannabis use: 24.31 [23.98, 24.65], females with cannabis use: 19.25 [19.05, 19.45]; Table 2). NH Asian or Pacific Islander students had the lowest prevalence in both cohorts, while NH American Indian or Alaskan Native (AI/AN) students had the highest prevalence in the overall cohort. Among students with lifetime cannabis use, NH AI/AN, NH Other, and NH Multiracial students had the highest prevalence. In both cohorts, undergraduate students had higher prevalence than Master’s/PhD students, temporary/unhoused students and students in fraternities had higher prevalence than other housing situations, and students in the western U.S. region had higher prevalence than other regions. Table 3 presents the distribution of cannabis involvement severity among those with lifetime cannabis use: 7.32% (95% CI: 7.21, 7.43) had mild involvement, 6.88% (95% CI: 6.78, 6.99) had moderate involvement, and 6.56% (95% CI: 6.46, 6.67) had severe/high involvement.

**Table 2:** Prevalence of elevated problematic cannabis involvement (score ≥ 2) among all students and those with lifetime cannabis use^1^ overall and stratified by characteristics

Outcome/cohort	Elevated problematic cannabis involvement among all students		Elevated problematic cannabis involvement among students with lifetime cannabis use	
Characteristic	n (%^2^)	95% CI^3^	n (%)	95% CI
Overall	46,308 (8.66)	8.58–8.73	45,503 (20.77)	20.60–20.94
Age				
18–19	10,830 (7.13)	7.00–7.26	10,641 (21.50)	21.14–21.86
20	7,566 (9.44)	9.23–9.64	7,446 (22.63)	22.18–23.08
21–24	17,641 (10.40)	10.25–10.54	17,386 (22.69)	22.39–22.98
≥25	9,800 (7.72)	7.57–7.87	9,634 (16.46)	16.16–16.76
Sex				
Female	30,075 (8.18)	8.10–8.27	29,707 (19.25)	19.05–19.45
Male	15,973 (9.69)	9.55–9.84	15,580 (24.31)	23.98–24.65
Race/ethnicity				
AI/AN	1,478 (12.71)	12.10–13.31	1,425 (26.93)	25.74–28.13
Hispanic	7,054 (8.57)	8.38–8.76	6,943 (20.62)	20.18–21.05
NH API	2,881 (3.70)	3.57–3.83	2,741 (15.34)	14.82–15.87
NH Black	2,124 (8.08)	7.75–8.41	2,046 (23.05)	22.17–23.93
NH White	28,383 (9.68)	9.58–9.79	28,107 (20.74)	20.52–20.96
NH Other	1,128 (7.68)	7.25–8.11	1,048 (25.14)	23.82–26.45
NH Multiracial	3,260 (11.29)	10.92–11.65	3,193 (23.30)	22.60–24.01
Academic year				
Undergraduate	37,817 (9.43)	9.34–9.52	37,199 (22.94)	22.74–23.15
Master’s or PhD	7,670 (6.18)	6.05–6.32	7,531 (14.17)	13.88–14.47
Other/not seeking degree	570 (7.87)	7.25–8.49	564 (17.66)	16.34–18.99
Housing status				
Fraternity	980 (14.27)	13.44–15.10	940 (27.18)	25.69–28.66
On-campus	12,818 (7.12)	7.00–7.23	12,617 (19.51)	19.20–19.81
Family	6,633 (6.59)	6.44–6.75	6,508 (20.60)	20.15–21.05
Off-campus	24,763 (10.49)	10.37–10.61	24,428 (21.22)	20.98–21.46
Temp/unhoused	474 (18.65)	17.13–20.16	429 (34.85)	32.19–37.51
Other	377 (6.16)	5.56–6.76	371 (15.60)	14.14–17.06
Region				
NE	9,359 (8.26)	8.10–8.42	9,195 (18.60)	18.25–18.94
MW	9,417 (7.74)	7.59–7.89	9,287 (19.36)	19.01–19.72
S	11,691 (7.79)	7.66–7.93	11,411 (20.88)	20.54–21.22
W	15,841 (10.57)	10.41–10.73	15,610 (23.29)	22.97–23.61

1 lifetime cannabis use defined by students answering in the affirmative to the question: “In your life, which of the following substances have you ever used: Cannabis (marijuana, weed, hash, edibles, vaped cannabis, etc.) [Please report nonmedical use only.]” 2 row % 3 confidence interval

**Table 3:** Distribution of cannabis involvement severity levels among students with lifetime cannabis use overall and stratified by characteristics

Characteristic	Mild involvement		Moderate involvement		Severe/high involvement	
	*n* (%^1^)	95% CI^2^	*n* (%^1^)	95% CI^2^	*n* (%^1^)	95% CI^2^
Overall	16,044 (7.32)	7.21–7.43	15,079 (6.88)	6.78–6.99	14,380 (6.56)	6.46–6.67
Age						
18–19	4,123 (8.33)	8.09–8.57	3,204 (6.47)	6.26–6.69	3,314 (6.70)	6.48–6.92
20	2,502 (7.60)	7.32–7.89	2,373 (7.21)	6.93–7.49	2,571 (7.81)	7.52–8.10
21–24	5,802 (7.57)	7.38–7.76	5,753 (7.51)	7.32–7.69	5,831 (7.61)	7.42–7.80
≥25	3,471 (5.93)	5.74–6.12	3,622 (6.19)	5.99–6.38	2,541 (4.34)	4.18–4.51
Sex						
Female	10,491 (6.80)	6.67–6.92	10,131 (6.56)	6.44–6.69	9,085 (5.89)	5.77–6.00
Male	5,477 (8.55)	8.33–8.76	4,880 (7.62)	7.41–7.82	5,223 (8.15)	7.94–8.36
Race/ethnicity						
AI/AN	482 (9.11)	8.33–9.89	501 (9.47)	8.68–10.26	442 (8.35)	7.61–9.10
Hispanic	2,522 (7.49)	7.21–7.77	2,385 (7.08)	6.81–7.36	2,036 (6.05)	5.79–6.30
NH API	1,124 (6.29)	5.94–6.65	871 (4.88)	4.56–5.19	746 (4.18)	3.88–4.47
NH Black	732 (8.25)	7.67–8.82	698 (7.86)	7.30–8.42	616 (6.94)	6.41–7.47
NH White	9,772 (7.21)	7.07–7.35	9,305 (6.79)	6.66–6.93	9,130 (6.74)	6.60–6.87
NH Other	382 (9.16)	8.29–10.04	330 (7.92)	7.10–8.74	336 (8.06)	7.23–8.89
NH Multiracial	1,030 (7.52)	7.08–7.96	1,089 (7.95)	7.49–8.40	1,074 (7.84)	7.39–8.29
Academic year						
Undergraduate	12,905 (7.96)	7.83–8.09	12,036 (7.42)	7.30–7.55	12,258 (7.56)	7.43–7.69
Master’s or PhD	2,857 (5.38)	5.19–5.57	2,769 (5.21)	5.02–5.40	1,905 (3.59)	3.43–3.74
Other/not seeking degree	206 (6.45)	5.60–7.30	204 (6.39)	5.54–7.24	154 (4.82)	4.08–5.57
Housing status						
Fraternity	307 (8.88)	7.93–9.82	293 (8.47)	7.54–9.40	340 (9.83)	8.84–10.82
On-campus	4,833 (7.47)	7.27–7.68	3,928 (6.07)	5.89–6.26	3,856 (5.96)	5.78–6.14
Family	2,322 (7.35)	7.06–7.64	2,131 (6.75)	6.47–7.02	2,055 (6.50)	6.23–6.78
Off-campus	8,230 (7.15)	7.00–7.30	8,385 (7.28)	7.13–7.43	7,813 (6.79)	6.64–6.93
Temp/unhoused	149 (12.10)	10.28–13.93	132 (10.72)	8.99–12.45	148 (12.02)	10.21–13.84
Other	129 (5.42)	4.51–6.34	150 (6.31)	5.33–7.28	92 (3.87)	3.09–4.64
Region						
NE	3,187 (6.45)	6.23–6.66	3,191 (6.45)	6.24–6.67	2,817 (5.70)	5.49–5.90
MW	3,415 (7.12)	6.89–7.35	2,861 (5.97)	5.75–6.18	3,011 (6.28)	6.06–6.50
S	4,185 (7.66)	7.43–7.88	3.541 (6.48)	6.27–6.69	3,685 (6.74)	6.53–6.95
W	5,257 (7.84)	7.64–8.05	5,486 (8.18)	7.98–8.39	4,867 (7.26)	7.06–7.46

1 row % 2 confidence interval

### Inferential statistics

**Fig. 2:**
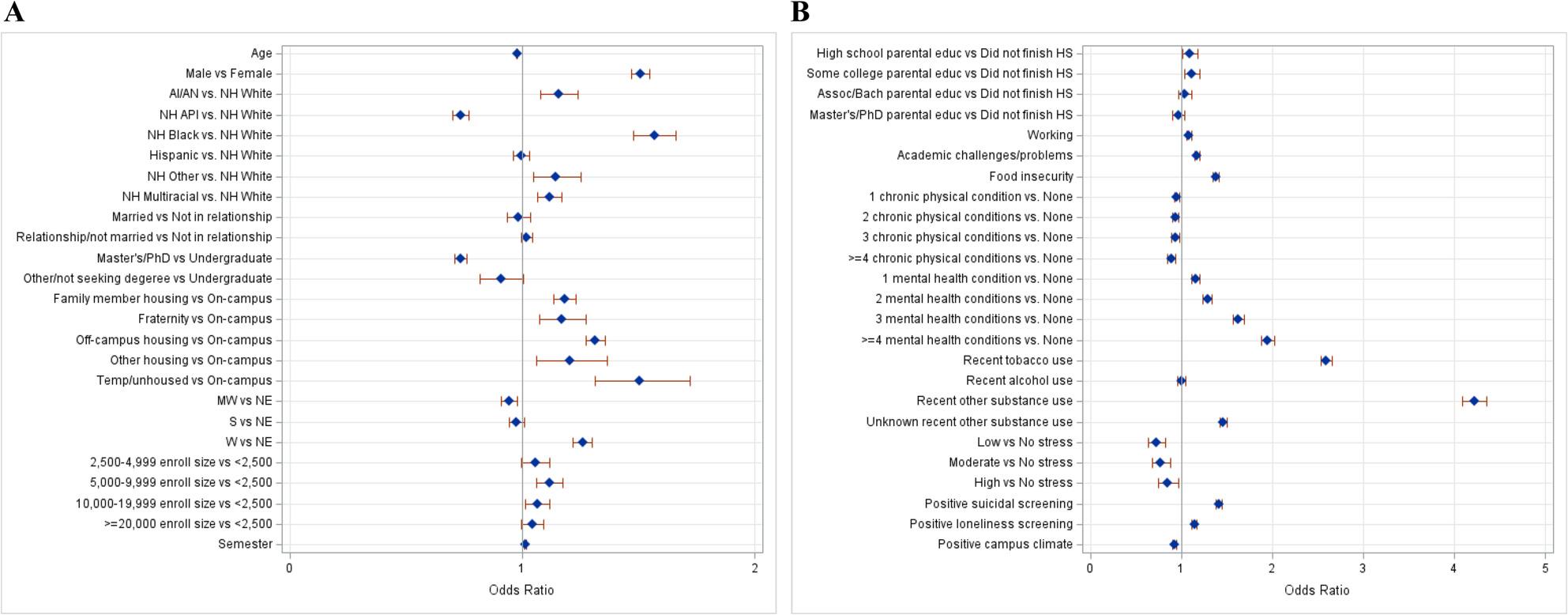
**A**. Characteristic associations with cannabis involvement severity among students with lifetime cannabis use. Tabularized values in Supplemental Table 2. **B**. Characteristic associations with cannabis involvement severity among students with lifetime cannabis use. Tabularized values in Supplemental Table 2

Figure 2A and B display aORs (95% error bars) and Supplemental Table 2 displays tabularized aORs (95% CIs) for associations between student characteristics and cannabis involvement severity among those with lifetime cannabis use. There was a higher likelihood of greater involvement severity for males (aOR [95% CI]: 1.51 [1.47, 1.55]), AI/AN (1.16 [1.08, 1.24]), NH Black (1.57 [1.48, 1.66]), NH Other (1.15 [1.05, 1.25]), and NH multiracial students (1.12 [1.07, 1.17]; all compared to NH White). A higher likelihood of greater involvement severity was also observed for all non-campus housing situations (with the highest odds in temporary/unhoused students [1.50 [1.31, 1.72]] compared with on-campus), students in the western U.S. region (1.26 [1.22, 1.30]; compared with the northeast), students with academic challenges (1.17 [1.15, 1.20]), students reporting food insecurity (1.38 [1.34, 1.42]), students with greater counts of chronic mental health conditions (≥ 4: 1.95[1.88, 2.02]), recent tobacco (2.60 [2.53, 2.66]) and other substance use (4.22 [4.09, 4.35]), and students positive for suicidal (1.42 [1.38, 1.45]) or loneliness screening (1.14 [1.11, 1.17]). There was a lower likelihood of greater involvement severity for NH API students (0.73 [0.70, 0.77]; compared with NH White), Master’s/PhD students (0.74 [0.71, 0.76]; compared with undergraduates), students with greater counts of chronic physical conditions (≥ 4: 0.89 [0.85, 0.93]), those reporting higher stress levels (High: 0.85 [0.75, 0.97]), and those reporting a positive campus climate (0.92 [0.90, 0.95]).

Table 4 displays associations between cannabis involvement severity and binary mental health diagnosed conditions among students with lifetime cannabis use. Compared to students with no/low cannabis involvement, those with moderate (aPR [95% CI]: 1.03 [1.01, 1.06]) and severe/high (1.04 [1.02, 1.07]) involvement had higher prevalence of anxiety. Those with mild (1.05 [1.02, 1.08]), moderate (1.07 [1.04, 1.10]), and severe/high (1.08 [1.05, 1.11]) involvement had higher prevalence of depression. Those with mild (1.07 [1.04, 1.10]), moderate (1.10 [1.07, 1.13]), and severe/high (1.07 [1.04, 1.10]) involvement had higher prevalence of other mental health conditions. Those with mild (1.03 [1.01, 1.05]) and severe/high (1.04 [1.02, 1.07]) involvement had higher prevalence of mental health service receipt.

**Table 4:** Associations between cannabis involvement severity and mental health diagnosed conditions among students with lifetime cannabis use

Outcome	Diagnosed conditions						Receipt of mental health services	
	Anxiety		Depression		Other			
Cannabis involvement severity	n (%^1^)	aPR^2^ (95% CI^3^)	n (%^1^)	aPR^2^ (95% CI^3^)	n (%^1^)	aPR^2^ (95% CI^3^)	n (%^1^)	aPR^2^ (95% CI^3^)
No/low	66,810 (38.71)	1 [Ref]	54,611 (31.67)	1 [Ref]	47,741 (29.57)	1 [Ref]	107,874 (62.28)	1 [Ref]
Mild involvement	7,505 (47.20)	1.01 (0.99, 1.04)	6,832 (43.00)	1.05 (1.02, 1.08)	6,076 (40.25)	1.07 (1.04, 1.10)	10,891 (68.15)	1.03 (1.01, 1.05)
Moderate involvement	7,600 (50.80)	1.03 (1.01, 1.06)	6,919 (46.30)	1.07 (1.04, 1.10)	6,168 (43.37)	1.10 (1.07, 1.13)	10,499 (69.86)	*1.02 (1.00*,* 1.04)*
Severe/high involvement	7,981 (55.92)	1.04 (1.02, 1.07)	7,573 (53.13)	1.08 (1.05, 1.11)	6,566 (48.34)	1.07 (1.04, 1.10)	10,653 (74.27)	1.04 (1.02, 1.07)
Mild involvement	7,505 (47.20)	1 [Ref]	6,832 (43.00)	1 [Ref]	6,076 (40.25)	1 [Ref]	10,891 (68.15)	1 [Ref]
Moderate involvement	7,600 (50.80)	1.02 (0.99, 1.05)	6,919 (46.30)	1.02 (0.98, 1.05)	6,168 (43.37)	1.03 (0.99, 1.06)	10,499 (69.86)	0.99 (0.96, 1.02)
Severe/high involvement	7,981 (55.92)	*1.03 (1.00*,* 1.06)*	7,573 (53.13)	1.03 (0.99, 1.06)	6,566 (48.34)	1.00 (1.97, 1.04)	10,653 (74.27)	1.01 (0.99, 1.04)
Moderate involvement	7,600 (50.80)	1 [Ref]	6,919 (46.30)	1 [Ref]	6,168 (43.37)	1 [Ref]	10,499 (69.86)	1 [Ref]
Severe/high involvement	7,981 (55.92)	1.01 (0.98, 1.04)	7,573 (53.13)	1.01 (0.98, 1.05)	6,566 (48.34)	0.98 (0.94, 1.01)	10,653 (74.27)	*1.03 (1.00*,* 1.05)*

**1** row % **2** adjusted prevalence ratio via modified Poisson regression; bolded values represent statistically significant results (*p* < 0.05), italicized values represent results on the boundary of statistical significance (0.05 ≤ *p* < 0.10) **3** confidence interval

Table 5 displays associations between cannabis involvement severity and mental health constructs among those with lifetime cannabis use. Increasing involvement severity, compared to no/low involvement, was associated with higher non-specific serious mental illness (mild $\:{e}^{{\widehat{\beta\:}}_{ADJ}}$ [95% CI]: 1.08 [1.07, 1.09]; moderate: 1.09 [1.08, 1.10]; severe/high: 1.14 [1.12, 1.15]), loneliness (mild: 1.02 [1.02, 1.03]; moderate: 1.02 [1.01, 1.02]; severe/high: 1.05 [1.05, 1.06]), and suicide (mild: 1.08 [1.07, 1.09]; moderate: 1.07 [1.06, 1.08]; severe/high: 1.14 [1.13, 1.15]), screening scores. Severe/high involvement, compared to no/low involvement, was associated with lower flourishing (0.96 [0.95, 0.97]) and lower resilience (0.96 [0.95, 0.97]). Severe/high involvement was also associated with higher percent mental illness/loneliness/suicide scores and lower percent flourishing/resilience scores when compared to both mild and moderate involvement groups.

**Table 5:** Associations between cannabis involvement severity and mental health constructs among students with lifetime cannabis use

Outcome	Constructs									
	Non-specific serious mental illness		Loneliness		Suicide		Flourishing		Resilience	
Cannabis involvement severity	Median (IQR^1^)	$\:{\boldsymbol{e}}^{{\widehat{\boldsymbol{\beta\:}}}_{\boldsymbol{A}\boldsymbol{D}\boldsymbol{J}}2}$(95% CI^3^)	Median (IQR^1^)	$\:{\boldsymbol{e}}^{{\widehat{\boldsymbol{\beta\:}}}_{\boldsymbol{A}\boldsymbol{D}\boldsymbol{J}}2}$(95% CI^3^)	Median (IQR^1^)	$\:{\boldsymbol{e}}^{{\widehat{\boldsymbol{\beta\:}}}_{\boldsymbol{A}\boldsymbol{D}\boldsymbol{J}}2}$(95% CI^3^)	Median (IQR^1^)	$\:{\boldsymbol{e}}^{{\widehat{\boldsymbol{\beta\:}}}_{\boldsymbol{A}\boldsymbol{D}\boldsymbol{J}}2}$(95% CI^3^)	Median (IQR^1^)	$\:{\boldsymbol{e}}^{{\widehat{\boldsymbol{\beta\:}}}_{\boldsymbol{A}\boldsymbol{D}\boldsymbol{J}}2}$(95% CI^3^)
No/low	8.00 (5.00–12.00)	1 [Ref]	5.00 (4.00–7.00)	1 [Ref]	5.00 (3.00–7.000)	1 [Ref]	47.00 (41.00–51.00)	1 [Ref]	6.00 (5.00–7.00)	1 [Ref]
Mild involvement	10.00 (6.00–14.00)	1.08 (1.07, 1.09)	6.00 (4.00–7.00)	1.02 (1.02, 1.03)	6.00 (3.00–9.00)	1.08 (1.07, 1.09)	45.00 (38.00–49.00)	0.99 (0.98, 1.00)	6.00 (5.00–7.00)	0.99 (0.98, 1.00)
Moderate involvement	10.00 (6.00–14.00)	1.09 (1.08, 1.10)	6.00 (4.00–7.00)	1.02 (1.01, 1.02)	6.00 (3.00–9.00)	1.07 (1.06, 1.08)	46.00 (39.00–50.00)	0.99 (0.98, 1.00)	6.00 (5.00–7.00)	0.99 (0.98, 1.00)
Severe/high involvement	12.00 (8.00–15.00)	1.14 (1.12, 1.15)	6.00 (5.00–8.00)	1.05 (1.05, 1.06)	7.00 (5.00–11.00)	1.14 (1.13, 1.15)	43.00 (36.00–48.00)	0.96 (0.95, 0.97)	6.00 (5.00–7.00)	0.96 (0.95, 0.97)
Mild involvement	10.00 (6.00–14.00)	1 [Ref]	6.00 (4.00–7.00)	1 [Ref]	6.00 (3.00–9.00)	1 [Ref]	45.00 (38.00–49.00)	1 [Ref]	6.00 (5.00–7.00)	1 [Ref]
Moderate involvement	10.00 (6.00–14.00)	1.00 (0.99, 1.02)	6.00 (4.00–7.00)	0.99 (0.99, 1.00)	6.00 (3.00–9.00)	0.99 (0.98, 1.01)	46.00 (39.00–50.00)	1.01 (1.00, 1.02)	6.00 (5.00–7.00)	1.01 (1.00, 1.02)
Severe/high involvement	12.00 (8.00–15.00)	1.05 (1.04, 1.06)	6.00 (5.00–8.00)	1.03 (1.02, 1.04)	7.00 (5.00–11.00)	1.06 (1.05, 1.07)	43.00 (36.00–48.00)	0.98 (0.97, 0.99)	6.00 (5.00–7.00)	0.98 (0.97, 0.99)
Moderate involvement	10.00 (6.00–14.00)	1 [Ref]	6.00 (4.00–7.00)	1 [Ref]	6.00 (3.00–9.00)	1 [Ref]	46.00 (39.00–50.00)	1 [Ref]	6.00 (5.00–7.00)	1 [Ref]
Severe/high involvement	12.00 (8.00–15.00)	1.05 (1.03, 1.06)	6.00 (5.00–8.00)	1.04 (1.03, 1.04)	7.00 (5.00–11.00)	1.06 (1.05, 1.07)	43.00 (36.00–48.00)	0.97 (0.96, 0.98)	6.00 (5.00–7.00)	0.97 (0.96, 0.98)

1 interquartile range 2 exponentiated adjusted coefficient via log-linear regression; bolded values represent statistically significant results (*p* < 0.05), italicized values represent results on the boundary of statistical significance (0.05 ≤ *p* < 0.10); Kessler 6 R^2^: 0.40; UCLA R^2^: 0.24; SBQR R^2^: 0.29; Diener R^2^: 0.21; Connor-Davidson R^2^: 0.13 3 confidence interval

### Supplemental SUD/cannabis SSIS/cannabis involvement validation analysis

Rotated factor loadings, communalities, and inter-factor correlations are presented in Supplemental Tables 3a-3b from the exploratory factor analysis. The supplemental comparison of elevated problematic cannabis involvement (score ≥ 2) to the formal survey SUD question displayed a weak phi coefficient (*r*_*ϕ*_= 0.12) yet a more moderate tetrachoric correlation (*r*_*tet*_= 0.42). While cannabis involvement severity demonstrated a strong positive correlation with cannabis SSIS (*r*_*s*_= 0.76), the prevalence of moderate/high cannabis SSIS was more than double the prevalence of elevated problematic cannabis involvement among those with lifetime cannabis use (45.61% vs. 20.77%). The prevalence of high cannabis SSIS was 2.70% among those with cannabis use history (Supplemental Table 4). Further comparative analysis is presented in Supplemental Tables 5–6. Supplemental Table 5 presents characteristic associations with SUD and elevated problematic cannabis involvement in students overall. Associations aligned well except in cases of age, chronic physical conditions, and recent alcohol use. Age was associated with a higher likelihood of SUD (aOR [95% CI]: 1.06 [1.05, 1.07]) but a lower likelihood of elevated problematic cannabis involvement (aOR [95% CI]: 0.98 [0.97, 0.99]). Higher counts of chronic physical conditions were associated with higher likelihood of SUD but lower likelihood of elevated problematic cannabis involvement. Recent alcohol use was associated with lower likelihood of SUD (aOR [95% CI]: 0.29 [0.27, 0.31]) but higher likelihood of elevated problematic cannabis involvement (aOR [95% CI]: 2.76 [2.66, 2.87]). When comparing characteristic associations with elevated problematic cannabis involvement and with moderate/high cannabis SSIS, associations again aligned in most cases however diverged in other race/ethnicity, relationship status, all housing situations, and elevated stress levels. Supplemental Table 6 presents associations between SUD/moderate or high cannabis SSIS/elevated problematic cannabis involvement and binary mental health indicators, and associations aligned across all outcomes.

## Discussion

Among a comprehensive sample of U.S. college students, this study estimated the prevalence and severity distribution of problematic cannabis involvement using cannabis-specific ASSIST items embedded within the ACHA-NCHA III. We identified characteristics associated with greater involvement severity among students with lifetime cannabis use. Lastly, we identified graded associations between involvement severity and multiple mental health indicators again among those with cannabis use history.

Among all U.S. college students, the prevalence of elevated problematic cannabis involvement (score ≥ 2) was 8.66%, and prevalence was substantially higher (20.77%) among the sub-sample of students with lifetime cannabis use. Although these estimates reflect a screening-oriented involvement index rather than a DSM-5 cannabis use disorder diagnosis, the observed magnitude is broadly consistent with prior work indicating that a meaningful minority of college-age or college-enrolled students that engage in cannabis use experience clinically concerning use-related behaviors and consequences (Caldeira et al. 2008; Choi et al. 2024; Hasin et al. 2015; Morissette et al. 2024).

Elevated problematic cannabis involvement varied across sociodemographic groups. Across the overall student cohort, the oldest students (≥ 25) had the lowest prevalence, and males had higher prevalence than females. NH API students consistently had the lowest prevalence, while NH AI/AN and NH multiracial students had among the highest prevalence, aligning with prior evidence of heterogeneity in cannabis-related risk across demographic populations and cannabis-use subgroups (Choi et al. 2024; Hasin et al. 2015; Keith et al. 2015; Phillips et al. 2018). Undergraduates and students living off campus or in temporary/unhoused settings also demonstrated higher prevalence compared with graduate students and those living on campus. The lower prevalence observed among older-aged and graduate students may be explained by a possible “maturing out” phenomenon that has been observed in alcohol use. Around age 22 major reductions in problematic drinking habits have been reported possibly due to assuming more responsibilities of adulthood, changing attitudes toward drinking, and personality maturation (Lee and Sher 2018; O’Malley 2004).

Consistent with these patterns, our multivariable analyses among those with cannabis use history found that male sex, several racial/ethnic minoritized groups (including AI/AN and NH Black), non-campus housing, undergraduate status, academic challenges, food insecurity, greater co-occurring substance use, and greater counts of chronic mental health conditions were associated with higher odds of more severe problematic cannabis involvement. These correlates likely reflect overlapping structural, psychosocial, and behavioral vulnerabilities that co-occur with harmful substance involvement in college settings, including housing instability and related adversity (Alhammad et al. 2022; Ebling et al. 2025; Lui et al. 2023; McKinnon et al. 2023; Wolitzky-Taylor et al. 2017).

Changes in cannabis product availability and potency may contribute to the observed burden of problematic involvement among college students. In legal markets, cannabis flower products commonly exhibit high THC concentrations, and concentrates can reach substantially higher potencies (MPG Consulting & University of Colorado Boulder Leeds School of Business, 2021; Smart et al. 2017; Vergara et al. 2017). Emerging evidence suggests that higher-potency cannabis exposure is associated with increased risk of cannabis-related problems and adverse mental health outcomes (Barrington-Trimis et al. 2020; Hammond et al. 2020; Petrilli et al. 2022).

Our findings also indicate a possible dose-response relationship between cannabis involvement severity and multiple mental health indicators among students with lifetime cannabis use. Higher involvement severity was associated with greater prevalence of anxiety, depression, and other mental health conditions, higher utilization of mental health services, and worse scores on non-specific serious mental illness, loneliness, and suicide behavior screening measures. These results align with the broader literature documenting strong co-occurrence between problematic cannabis use patterns and mental health burden in young adults (Ferland and Hurd 2020; Hasin et al. 2016; Kedzior and Laeber 2014; Rhew et al. 2025; Sultan et al. 2023).

From a clinical and campus health perspective, more severe patterns of problematic cannabis involvement may serve as a practical flag for more comprehensive screening of co-occurring mental health concerns and other substance use, as well as targeted prevention and early intervention strategies. Prior work suggests that coping-oriented motives and self-medication processes are strongly linked to cannabis-related problems, supporting integrated behavioral health approaches within campus health systems (Brodbeck et al. 2007; Buckner 2013; Glodosky and Cuttler 2020). Future work should examine longitudinal ordering of cannabis involvement and mental health symptoms and identify modifiable mechanisms (e.g., coping motives, sleep disruption, or academic stress) that could be targeted in campus-based services.

### Limitations

While this study provides valuable evidence from a large national multi-institution sample of U.S. college students, several limitations should be considered. First, the ACHA-NCHA III is cross-sectional, preventing causal inference. Second, all responses are self-reported and may be subject to recall or social desirability bias. Third, respondents are students enrolled in U.S. colleges or universities, and results may not generalize to non-college young adults. Although the ACHA-NCHA III spans a large number of institutions nationwide, it is not designed to yield nationally representative estimates of all U.S. college students; prevalence estimates should be interpreted as patterns within this multi-campus sample. Fourth, our primary outcome is a screening-based cannabis involvement index derived from ASSIST-related items rather than a formal DSM-5 cannabis use disorder diagnosis; thus, prevalence estimates should be interpreted as elevated problematic involvement rather than diagnostic CUD prevalence. Finally, the survey does not capture key use characteristics such as cannabis product type, potency, and use motives, which may influence both problematic use patterns and mental health indicators.

## Conclusions

In conclusion, elevated problematic cannabis involvement is common among U.S. college students and is patterned by demographic, social, and clinical characteristics. Among students with lifetime cannabis use, greater involvement severity is associated with substantially higher mental health burden across multiple domains. These findings support integrating cannabis-use screening with mental health screening and expanding targeted prevention and intervention strategies within campus health systems.

## Supplementary Information

Supplementary Material 1.

## Data Availability

The data that support the findings of this study are available from the American College Health Association National College Health Assessment (ACHA-NCHA) and are not publicly available. Data can be requested directly from ACHA (contact: Christine Kukich, [ckukich@acha.org](mailto: ckukich@acha.org) ) under a data use agreement.
