# Supplementary material for: Operationalizing ASSIST-based indicators to estimate the prevalence and correlates of problematic cannabis involvement among U.S. college students
Source: J Cannabis Res. 2026 May 14;8:81. doi: 10.1186/s42238-026-00446-4 (PMC13339261; doi:10.1186/s42238-026-00446-4)
Supplement: Supplementary file 1 — Supplementary Material 1. [file 42238_2026_446_MOESM1_ESM.docx]

**Supplemental Table 1.** Cannabis involvement indicators derived from ACHA-NCHA III (ASSIST module) and scoring approach

| Cannabis involvement domain | NCHA III (ASSIST module) Variable | Scoring^1^ |
| --- | --- | --- |
| Use frequency (past 3 months) | **N3Q22B3** In the past 3 months, how often have you used the substance(s) you  mentioned? (rows endorsed in **N3Q22A** are displayed for this question) | Never (0), Once or twice (2), Monthly (3), Weekly (4), **Daily or almost daily (6)** |
| Unsuccessful attempts to cut down/stop | **N3Q22O3** Have you ever tried and failed to control, cut down or stop using the following  substance(s)? (rows endorsed in **N3Q22A** are displayed for this question) | No, never (0); **Yes, in the past 3 months (6); Yes, but not in the past 3 months (3)** |
| Time/engagement with cannabis use (proxy) | **N3Q22B3** In the past 3 months, how often have you used the substance(s) you  mentioned? (rows endorsed in **N3Q22A** are displayed for this question)  **or**  **N3Q32** Do you identify as a person in recovery from alcohol or other drug use? | Never (0), Once or twice (2), Monthly (3), Weekly (4), **Daily or almost daily (6)**  **or**  No (1)  **Yes. Please specify the type of substance: (2) N3Q32TEXT= “CANNABIS”** |
| Craving/urge | **N3Q22K3** During the past 3 months, how often have you had a strong desire or urge to use the following substance(s)? (rows endorsed in **N3Q22B** are displayed for this question) | Never (0), Once or twice (3), Monthly (4**), Weekly (5), Daily or almost daily (6)** |
| Role impairment (missed obligations) | **N3Q22M3** During the past 3 months, how often have you failed to do what was normally  expected of you because of your use of the following substance(s)? (rows endorsed in  **N3Q22B** are displayed for this question)  **or**  (if they select that they used cannabis within the last 12 months in **N3Q24**)  **N3Q31B** Within the last 12 months, to what extent did your cannabis/marijuana use affect your academic performance? (Please select the most serious outcome below) | Never (0), Once or twice (5), **Monthly (6), Weekly (7), Daily or almost daily (8)**  **or**  My cannabis/marijuana use did not affect my academics (1)  **My cannabis/marijuana use negatively impacted my performance in a class (2)**  **My cannabis/marijuana use delayed progress towards my degree (3)** |
| Social/interpersonal concerns | **N3Q22N3** Has a friend or relative or anyone else ever expressed concern about your use  of the following substance(s)? (rows endorsed in **N3Q22A** are displayed for this question)  **or**  **N3Q22L3** During the past 3 months, how often has your use of the following  substance(s) led to health, social, legal, or financial problems? (rows endorsed in **N3Q22B** are displayed for this question) | No, never (0); **Yes, in the past 3 months (6);** Yes, but not in the past 3 months (3)  **or**  Never (0), Once or twice (4), **Monthly (5), Weekly (6), Daily or almost daily (7)** |
| Reduced engagement in activities (academic impact proxy) | (if they select that they used cannabis within the last 12 months in **N3Q24**)  **N3Q31B** Within the last 12 months, to what extent did your cannabis/marijuana use affect  your academic performance? (Please select the most serious outcome below) | My cannabis/marijuana use did not affect my academics (1)  **My cannabis/marijuana use negatively impacted my performance in a class (2)**  **My cannabis/marijuana use delayed progress towards my degree (3)** |
| Hazardous use (e.g., driving after use) | (if they select that they used cannabis within the last 30 days in **N3Q24**)  **N3Q31A** Within the last 30 days, did you drive within 6 hours of using  cannabis/marijuana? | No (1)  **Yes (2)** |
| Continued use despite problems | **N3Q22L3** During the past 3 months, how often has your use of the following  substance(s) led to health, social, legal, or financial problems? (rows endorsed in **N3Q22B**  are displayed for this question) | Never (0), Once or twice (4), **Monthly (5), Weekly (6), Daily or almost daily (7)** |
| Tolerance | **N3Q22B3** In the past 3 months, how often have you used the substance(s) you  mentioned? (rows endorsed in **N3Q22A** are displayed for this question) | Never (0), Once or twice (2), Monthly (3), Weekly (4), **Daily or almost daily (6)** |
| Withdrawal symptoms | (  **N3Q22B3 (cannabis)** In the past 3 months, how often have you used the substance(s) you  mentioned? (rows endorsed in **N3Q22A** are displayed for this question)  **or**  **N3Q24** When, if ever, was the last time you used cannabis/marijuana? Please include  medical and non-medical use.  )  **and**  (  **N3Q22B2 (alcohol)** In the past 3 months, how often have you used the substance(s) you  mentioned? (rows endorsed in **N3Q22A** are displayed for this question)  **or**  **N3Q22B1 (tobacco)** In the past 3 months, how often have you used the substance(s) you  mentioned? (rows endorsed in **N3Q22A** are displayed for this question)  ) | (  Never (0), **Once or twice (2), Monthly (3), Weekly (4), Daily or almost daily (6)**  **or**  Never (1)  **Within the last 2 weeks (2)**  **More than 2 weeks ago but within the last 30 days (3)**  **More than 30 days ago but within the last 3 months (4)**  More than 3 months ago but within the last 12 months (5)  More than 12 months ago (6)  )  **and**  (  Never (0), Once or twice (2), Monthly (3), Weekly (4), **Daily or almost daily (6)**  **or**  Never (0), Once or twice (2), Monthly (3), Weekly (4), **Daily or almost daily (6)**  ) |

**1** The ACHA-NCHA III includes cannabis-specific items from the ASSIST module capturing domains such as use frequency, craving/urge, difficulty controlling use, role impairment, and cannabis-related problems. We operationalized a screening-based cannabis involvement severity index by dichotomizing 11 indicator items (1=endorsed) and summing indicators (range 0-11). Participants meeting each scoring criterion (**bolded response categories**) were flagged as 1; all others were flagged as 0. Scores were categorized as no/low involvement (0-1), mild involvement (2-3), moderate involvement (4-5), and severe/high involvement (≥6); scores ≥2 represent elevated problematic cannabis involvement. **Numbers in parentheses reflect the original ASSIST response-option codes shown for transparency and were not used as numeric weights.**

**Supplemental Table 2.** Associations between characteristics and cannabis involvement severity among students with lifetime cannabis use

|  | Cannabis involvement severity |
| --- | --- |
| Predictor | **aOR^1^ (95% CI^2^)** |
| Age | **0.98 (0.97, 0.99)** |
| Sex |  |
| Female | 1 [Ref] |
| Male | **1.51 (1.47, 1.55)** |
| Race/ethnicity |  |
| NH White | 1 [Ref] |
| AI/AN | **1.16 (1.08, 1.24)** |
| Hispanic | 1.00 (0.96, 1.03) |
| NH API | **0.73 (0.70, 0.77)** |
| NH Black | **1.57 (1.48, 1.66)** |
| NH Other | **1.15 (1.05, 1.25)** |
| NH Multiracial | **1.12 (1.07, 1.17)** |
| Relationship status |  |
| Not in a relationship | 1 [Ref] |
| Relationship, not partnered/married | *1.02 (1.00, 1.05)* |
| Married/partnered | 0.99 (0.94, 1.04) |
| Academic year |  |
| Undergraduate | 1 [Ref] |
| Master’s or PhD | **0.74 (0.71, 0.76)** |
| Other/not seeking degree | *0.91 (0.82, 1.01)* |
| Housing status |  |
| On-campus | 1 [Ref] |
| Fraternity | **1.17 (1.07, 1.27)** |
| Family | **1.18 (1.14, 1.23)** |
| Off-campus | **1.32 (1.28, 1.36)** |
| Temp/unhoused | **1.50 (1.31, 1.72)** |
| Other | **1.21 (1.06, 1.37)** |
| Region |  |
| NE | 1 [Ref] |
| MW | **0.95 (0.91, 0.98)** |
| S | *0.98 (0.94, 1.01)* |
| W | **1.26 (1.22, 1.30)** |
| Total enrollment |  |
| <2,500 students | 1 [Ref] |
| 2,500-4,999 students | *1.06 (1.00, 1.12)* |
| 5,000-9,999 students | **1.12 (1.06, 1.18)** |
| 10,000-19,999 students | **1.07 (1.01, 1.12)** |
| ≥20,000 students | *1.04 (1.00, 1.09)* |
| Semester | **1.02 (1.01, 1.02)** |
| Parent educational attainment |  |
| Did not finish high school | 1 [Ref] |
| High school or GED | **1.10 (1.02, 1.18)** |
| Some college | **1.12 (1.04, 1.21)** |
| Associates/Bachelor’s | 1.04 (0.97, 1.11) |
| Master’s/PhD | 0.97 (0.90, 1.04) |
| Working |  |
| No | 1 [Ref] |
| Yes | **1.09 (1.06, 1.11)** |
| Problems or challenges with academics |  |
| No | 1 [Ref] |
| Yes | **1.17 (1.15, 1.20)** |
| Food insecurity |  |
| No | 1 [Ref] |
| Yes | **1.38 (1.34, 1.42)** |
| Chronic physical conditions |  |
| 0 | 1 [Ref] |
| 1 | **0.95 (0.92, 0.98)** |
| 2 | **0.93 (0.90, 0.97)** |
| 3 | **0.94 (0.89, 0.98)** |
| ≥4 | **0.89 (0.85, 0.93)** |
| Chronic mental health conditions |  |
| 0 | 1 [Ref] |
| 1 | **1.16 (1.12, 1.20)** |
| 2 | **1.29 (1.24, 1.34)** |
| 3 | **1.63 (1.57, 1.69)** |
| ≥4 | **1.95 (1.88, 2.02)** |
| Recent tobacco use |  |
| No | 1 [Ref] |
| Yes | **2.60 (2.53, 2.66)** |
| Recent alcohol use |  |
| No | 1 [Ref] |
| Yes | 1.00 (0.96, 1.04) |
| Recent other substance use |  |
| No | 1 [Ref] |
| Yes | **4.22 (4.09, 4.35)** |
| Unknown | **1.46 (1.42, 1.50)** |
| Recent stress levels |  |
| No stress | 1 [Ref] |
| Low | **0.73 (0.64, 0.83)** |
| Moderate | **0.77 (0.68, 0.88)** |
| High | **0.85 (0.75, 0.97)** |
| Positive for suicide screening |  |
| No | 1 [Ref] |
| Yes | **1.42 (1.38, 1.45)** |
| Positive for loneliness screening |  |
| No | 1 [Ref] |
| Yes | **1.14 (1.11, 1.17)** |
| Positive campus climate |  |
| No | 1 [Ref] |
| Yes | **0.92 (0.90, 0.95)** |

**1** adjusted odds ratio via ordinal logistic regression (proportional odds confirmed via plotting of empirical cumulative logits and maintaining parallel trends); bolded values represent statistically significant results (*p* < 0.05), italicized values represent results on the boundary of statistical significance (0.05 ≤ *p* < 0.10)*;* area under the receiver operating characteristic curve (AUROC) 0.77, via model trained on 90% of data and tested on 10% of randomly sampled hold-out data **2** confidence interval

**Supplemental Table 3a.** Rotated factor pattern loadings of ACHA-NCHA III cannabis involvement indicators

| NCHA Item | Factor 1 | Factor 2 | Factor 3 | Factor 4 | Communality |
| --- | --- | --- | --- | --- | --- |
| N3Q22B3 In the past 3 months, how often have you used the substance(s) you mentioned? | 0.90 | 0.31 | 0.46 | 0.34 | 0.82 |
| N3Q22O3 Have you ever tried and failed to control, cut down or stop using the following substance(s)? | 0.37 | 0.47 | 0.61 | 0.16 | 0.38 |
| N3Q32 Do you identify as a person in recovery from alcohol or other drug use? | 0.08 | 0.13 | 0.21 | 0.03 | 0.05 |
| N3Q22K3 During the past 3 months, how often have you had a strong desire or urge to use the following substance(s)? | 0.66 | 0.36 | 0.51 | 0.29 | 0.47 |
| N3Q22M3 During the past 3 months, how often have you failed to do what was normally expected of you because of your use of the following substance(s)? | 0.36 | 0.80 | 0.52 | 0.19 | 0.64 |
| N3Q31B Within the last 12 months, to what extent did your cannabis/marijuana use affect your academic performance? (Please select the most serious outcome below) | 0.24 | 0.60 | 0.46 | 0.11 | 0.37 |
| N3Q22N3 Has a friend or relative or anyone else ever expressed concern about your use of the following substance(s)? | 0.34 | 0.42 | 0.58 | 0.17 | 0.34 |
| N3Q22L3 During the past 3 months, how often has your use of the following  substance(s) led to health, social, legal, or financial problems? | 0.34 | 0.54 | 0.57 | 0.23 | 0.38 |
| N3Q31A Within the last 30 days, did you drive within 6 hours of using  cannabis/marijuana? | 0.52 | 0.29 | 0.40 | 0.20 | 0.29 |
| N3Q24 When, if ever, was the last time you used cannabis/marijuana? Please include medical and non-medical use. | -0.46 | -0.14 | -0.18 | -0.12 | 0.22 |
| N3Q22B2 (alcohol) In the past 3 months, how often have you used the substance(s) you mentioned? | 0.03 | 0.00 | -0.03 | 0.35 | 0.14 |
| N3Q22B1 (tobacco) In the past 3 months, how often have you used the substance(s) you mentioned? | 0.24 | 0.13 | 0.24 | 0.44 | 0.20 |

**1** iterated principal axis factor extraction with promax (power=3) rotation

**Supplemental Table 3b.** Inter-factor correlations of ACHA-NCHA III cannabis involvement indicators

|  | Factor 1 | Factor 2 | Factor 3 | Factor 4 |
| --- | --- | --- | --- | --- |
| Factor 1 | 1.00 | 0.39 | 0.56 | 0.37 |
| Factor 2 | 0.39 | 1.00 | 0.65 | 0.21 |
| Factor 3 | 0.56 | 0.65 | 1.00 | 0.32 |
| Factor 4 | 0.37 | 0.21 | 0.32 | 1.00 |

**Supplemental Table 4.** Prevalence of elevated problematic cannabis involvement (score ≥2) among all students and those with lifetime cannabis use, prevalence of moderate/high cannabis substance specific involvement score (SSIS; moderate/high ≥4; high ≥27) among those with lifetime cannabis use, overall and stratified by characteristics

|  | Elevated problematic cannabis involvement | | | | Moderate/high cannabis SSIS | | High cannabis SSIS | |
| --- | --- | --- | --- | --- | --- | --- | --- | --- |
| Outcome/cohort | **Among all students** | | **Among students with lifetime cannabis use** | | **Among students with lifetime cannabis use** | | **Among students with lifetime cannabis use** | |
| Characteristic | **n (%^1^)** | **95% CI^2^** | **n (%^1^)** | **95% CI^2^** | **n (%^1^)** | **95% CI^2^** | **n (%^1^)** | **95% CI^2^** |
| Overall | 46,308 (8.66) | 8.58 - 8.73 | 45,503 (20.77) | 20.60 - 20.94 | 96,885 (45.61) | 45.40 - 45.82 | 5,737 (2.70) | 2.63 - 2.77 |
| Age |  |  |  |  |  |  |  |  |
| 18-19 | 10,830 (7.13) | 7.00 **-** 7.26 | 10,641 (21.50) | 21.14 - 21.86 | 25,110 (52.83) | 52.38 - 53.28 | 1,502 (3.16) | 3.00 - 3.32 |
| 20 | 7,566 (9.44) | 9.23 **-** 9.64 | 7,446 (22.63) | 22.18 - 23.08 | 16,064 (50.60) | 50.05 - 51.15 | 1,091 (3.44) | 3.24 - 3.64 |
| 21-24 | 17,641 (10.40) | 10.25 **-** 10.54 | 17,386 (22.69) | 22.39 - 22.98 | 35,069 (47.19) | 46.83 - 47.55 | 2,202 (2.96) | 2.84 - 3.09 |
| ≥25 | 9,800 (7.72) | 7.57 **-** 7.87 | 9,634 (16.46) | 16.16 - 16.76 | 20,001 (34.84) | 34.45 - 35.23 | 833 (1.45) | 1.35 - 1.55 |
| Sex |  |  |  |  |  |  |  |  |
| Female | 30,075 (8.18) | 8.10 **-** 8.27 | 29,707 (19.25) | 19.05 - 19.45 | 67,415 (45.11) | 44.86 - 45.36 | 3,420 (2.29) | 2.21 - 2.36 |
| Male | 15,973 (9.69) | 9.55 **-** 9.84 | 15,580 (24.31) | 23.98 - 24.65 | 29,159 (46.76) | 46.37 - 47.16 | 2,250 (3.61) | 3.46 - 3.75 |
| Race/ethnicity |  |  |  |  |  |  |  |  |
| AI/AN | 1,478 (12.71) | 12.10 - 13.31 | 1,425 (26.93) | 25.74 - 28.13 | 2,587 (50.42) | 49.05 - 51.79 | 198 (3.86) | 3.33 - 4.39 |
| Hispanic | 7,054 (8.57) | 8.38 - 8.76 | 6,943 (20.62) | 20.18 - 21.05 | 15,130 (46.42) | 45.88 - 46.97 | 901 (2.76) | 2.59 - 2.94 |
| NH API | 2,881 (3.70) | 3.57 - 3.83 | 2,741 (15.34) | 14.82 - 15.87 | 6,961 (40.18) | 39.45 - 40.91 | 465 (2.68) | 2.44 - 2.92 |
| NH Black | 2,124 (8.08) | 7.75 - 8.41 | 2,046 (23.05) | 22.17 - 23.93 | 4,274 (50.19) | 49.13 - 51.25 | 293 (3.44) | 3.05 - 3.83 |
| NH White | 28,383 (9.68) | 9.58 - 9.79 | 28,107 (20.74) | 20.52 - 20.96 | 59,492 (45.21) | 44.94 - 45.48 | 3,241 (2.46) | 2.38 - 2.55 |
| NH Other | 1,128 (7.68) | 7.25 - 8.11 | 1,048 (25.14) | 23.82 - 26.45 | 1,901 (47.58) | 46.04 - 49.13 | 185 (4.63) | 3.98 - 5.28 |
| NH Multiracial | 3,260 (11.29) | 10.92 - 11.65 | 3,193 (23.30) | 22.60 - 24.01 | 6,540 (49.25) | 48.40 - 50.10 | 454 (3.42) | 3.11 - 3.73 |
| Academic year |  |  |  |  |  |  |  |  |
| Undergraduate | 37,817 (9.43) | 9.34 - 9.52 | 37,199 (22.94) | 22.74 - 23.15 | 77,791 (49.66) | 49.41 - 49.91 | 4,936 (3.15) | 3.06 - 3.24 |
| Master’s or PhD | 7,670 (6.18) | 6.05 - 6.32 | 7,531 (14.17) | 13.88 - 14.47 | 17,724 (34.06) | 33.65 - 34.46 | 678 (1.30) | 1.21 - 1.40 |
| Other/not seeking degree | 570 (7.87) | 7.25 - 8.49 | 564 (17.66) | 16.34 - 18.99 | 1,073 (34.24) | 32.58 - 35.90 | 69 (2.20) | 1.69 - 2.72 |
| Housing status |  |  |  |  |  |  |  |  |
| Fraternity | 980 (14.27) | 13.44 - 15.10 | 940 (27.18) | 25.69 - 28.66 | 1,807 (54.13) | 52.44 - 55.82 | 187 (5.60) | 4.82 - 6.38 |
| On-campus | 12,818 (7.12) | 7.00 - 7.23 | 12,617 (19.51) | 19.20 - 19.81 | 31,373 (50.35) | 49.95 - 50.74 | 1,808 (2.90) | 2.77 - 3.03 |
| Family | 6,633 (6.59) | 6.44 - 6.75 | 6,508 (20.60) | 20.15 - 21.05 | 13,288 (43.35) | 42.79 - 43.90 | 805 (2.63) | 2.45 - 2.81 |
| Off-campus | 24,763 (10.49) | 10.37 - 10.61 | 24,428 (21.22) | 20.98 - 21.46 | 48,744 (43.51) | 43.22 - 43.80 | 2,765 (2.47) | 2.38 - 2.56 |
| Temp/unhoused | 474 (18.65) | 17.13 - 20.16 | 429 (34.85) | 32.19 - 37.51 | 633 (53.42) | 50.58 - 56.26 | 85 (7.17) | 5.70 - 8.64 |
| Other | 377 (6.16) | 5.56 - 6.76 | 371 (15.60) | 14.14 - 17.06 | 742 (32.01) | 30.11 - 33.91 | 22 (0.95) | 0.01 - 1.43 |
| Region |  |  |  |  |  |  |  |  |
| NE | 9,359 (8.26) | 8.10 - 8.42 | 9,195 (18.60) | 18.25 - 18.94 | 22,157 (46.26) | 45.81 - 46.70 | 1,277 (2.67) | 2.52 - 2.81 |
| MW | 9,417 (7.74) | 7.59 - 7.89 | 9,287 (19.36) | 19.01 - 19.72 | 20,358 (43.74) | 43.29 - 44.19 | 1,135 (2.44) | 2.30 - 2.58 |
| S | 11,691 (7.79) | 7.66 - 7.93 | 11,411 (20.88) | 20.54 - 21.22 | 22,956 (43.32) | 42.90 - 43.74 | 1,458 (2.75) | 2.61 - 2.89 |
| W | 15,841 (10.57) | 10.41 - 10.73 | 15,610 (23.29) | 22.97 - 23.61 | 31,414 (48.34) | 47.95 - 48.72 | 1,867 (2.87) | 2.74 - 3.00 |

**1** row % **2** confidence interval

**Supplemental Table 5.** Associations between characteristics and SUD/moderate or high cannabis SSIS (≥4) /elevated problematic cannabis involvement (score ≥2), among all students

|  | SUD | Moderate/high cannabis SSIS | Elevated problematic cannabis involvement |
| --- | --- | --- | --- |
| Predictor | **aOR^1^ (95% CI^2^)** | **aOR (95% CI)** | **aOR (95% CI)** |
| Age | **1.06 (1.05, 1.07)** | **0.97 (0.96, 0.97)** | **0.98 (0.97, 0.99)** |
| Sex |  |  |  |
| Female | 1 [Ref] | 1 [Ref] | 1 [Ref] |
| Male | **2.08 (1.96, 2.20)** | **1.17 (1.14, 1.19)** | **1.51 (1.48, 1.55)** |
| Race/ethnicity |  |  |  |
| NH White | 1 [Ref] | 1 [Ref] | 1 [Ref] |
| AI/AN | **1.33 (1.17, 1.51)** | **1.14 (1.07, 1.21)** | **1.20 (1.12, 1.29)** |
| Hispanic | 1.06 (0.98, 1.15) | **1.08 (1.05, 1.11)** | 1.02 (0.99, 1.06) |
| NH API | **0.79 (0.70, 0.88)** | **0.86 (0.83, 0.89)** | **0.57 (0.55, 0.60)** |
| NH Black | 0.95 (0.82, 1.10) | **1.55 (1.47, 1.63)** | **1.58 (1.50, 1.68)** |
| NH Other | **0.82 (0.69, 0.99)** | **1.16 (1.07, 1.25)** | 0.99 (0.91, 1.08) |
| NH Multiracial | 0.98 (0.88, 1.09) | **1.11 (1.07, 1.15)** | **1.15 (1.10, 1.21)** |
| Relationship status |  |  |  |
| Not in a relationship | 1 [Ref] | 1 [Ref] | 1 [Ref] |
| Relationship, not partnered/married | 1.02 (0.96, 1.09) | **0.96 (0.94, 0.98)** | **1.11 (1.09, 1.14)** |
| Married/partnered | 0.99 (0.92, 1.08) | **0.87 (0.84, 0.90)** | 0.99 (0.94, 1.04) |
| Academic year |  |  |  |
| Undergraduate | 1 [Ref] | 1 [Ref] | 1 [Ref] |
| Master’s or PhD | **0.85 (0.79, 0.91)** | **0.76 (0.74, 0.78)** | **0.73 (0.71, 0.76)** |
| Other/not seeking degree | **0.83 (0.70, 0.99)** | **0.80 (0.73, 0.87)** | *0.91 (0.82, 1.01)* |
| Housing status |  |  |  |
| On-campus | 1 [Ref] | 1 [Ref] | 1 [Ref] |
| Fraternity | **1.48 (1.17, 1.86)** | 0.99 (0.91, 1.06) | **1.23 (1.13, 1.34)** |
| Family | **1.31 (1.20, 1.44)** | **0.81 (0.78, 0.84)** | **1.09 (1.05, 1.13)** |
| Off-campus | **1.53 (1.42, 1.65)** | **0.97 (0.94, 0.99)** | **1.37 (1.33, 1.41)** |
| Temp/unhoused | **2.28 (1.84, 2.83)** | 0.97 (0.85, 1.11) | **1.70 (1.49, 1.94)** |
| Other | **1.39 (1.15, 1.67)** | **0.87 (0.79, 0.97)** | 1.11 (0.98, 1.26) |
| Region |  |  |  |
| NE | 1 [Ref] | 1 [Ref] | 1 [Ref] |
| MW | **0.90 (0.82, 0.98)** | **0.85 (0.83, 0.88)** | **0.85 (0.82, 0.88)** |
| S | **0.85 (0.78, 0.92)** | **0.81 (0.78, 0.83)** | **0.85 (0.82, 0.88)** |
| W | **1.15 (1.06, 1.24)** | **1.17 (1.13, 1.20)** | **1.33 (1.28, 1.37)** |
| Total enrollment |  |  |  |
| <2,500 students | 1 [Ref] | 1 [Ref] | 1 [Ref] |
| 2,500-4,999 students | 0.99 (0.87, 1.13) | 1.03 (0.98, 1.08) | 1.02 (0.96, 1.08) |
| 5,000-9,999 students | 0.98 (0.87, 1.10) | 1.03 (0.98, 1.07) | **1.09 (1.04, 1.15)** |
| 10,000-19,999 students | 0.95 (0.85, 1.06) | 1.02 (0.98, 1.06) | **1.07 (1.01, 1.12)** |
| ≥20,000 students | 0.92 (0.83, 1.03) | *1.03 (100, 1.07)* | **1.07 (1.02, 1.12)** |
| Semester | **0.98 (0.97, 0.99)** | *1.01 (1.00, 1.02)* | *1.01 (1.00, 1.02)* |
| Parent educational attainment |  |  |  |
| Did not finish high school | 1 [Ref] | 1 [Ref] | 1 [Ref] |
| High school or GED | 1.05 (0.90, 1.21) | **1.10 (1.03, 1.16)** | **1.16 (1.08, 1.24)** |
| Some college | 1.05 (0.89, 1.22) | **1.09 (1.02, 1.16)** | **1.20 (1.11, 1.29)** |
| Associates/Bachelor’s | 1.01 (0.88, 1.17) | **1.06 (1.01, 1.13)** | **1.09 (1.02, 1.17)** |
| Master’s/PhD | 1.03 (0.89, 1.19) | *1.05 (0.99, 1.12)* | 1.04 (0.97, 1.12) |
| Working |  |  |  |
| No | 1 [Ref] | 1 [Ref] | 1 [Ref] |
| Yes | **1.12 (1.06, 1.19)** | **1.09 (1.07, 1.11)** | **1.13 (1.11, 1.16)** |
| Problems or challenges with academics |  |  |  |
| No | 1 [Ref] | 1 [Ref] | 1 [Ref] |
| Yes | **1.07 (1.01, 1.13)** | **1.09 (1.07, 1.11)** | **1.15 (1.12, 1.18)** |
| Food insecurity |  |  |  |
| No | 1 [Ref] | 1 [Ref] | 1 [Ref] |
| Yes | **1.11 (1.04, 1.18)** | **1.27 (1.24, 1.30)** | **1.37 (1.34, 1.41)** |
| Chronic physical conditions |  |  |  |
| 0 | 1 [Ref] | 1 [Ref] | 1 [Ref] |
| 1 | **1.21 (1.13, 1.31)** | **0.97 (0.95, 0.99)** | **0.96 (0.93, 0.98)** |
| 2 | **1.35 (1.25, 1.47)** | **0.95 (0.92, 0.98)** | **0.95 (0.91, 0.98)** |
| 3 | **1.38 (1.25, 1.51)** | **0.98 (0.94, 1.02)** | **0.93 (0.88, 0.97)** |
| ≥4 | **1.63 (1.49, 1.77)** | **0.90 (0.86, 0.94)** | **0.90 (0.86, 0.94)** |
| Chronic mental health conditions |  |  |  |
| 0 | 1 [Ref] | 1 [Ref] | 1 [Ref] |
| 1 | **3.25 (2.82, 3.74)** | **1.15 (1.12, 1.18)** | **1.33 (1.28, 1.37)** |
| 2 | **6.50 (5.66, 7.48)** | **1.22 (1.18, 1.26)** | **1.50 (1.44, 1.56)** |
| 3 | **10.24 (8.96, 11.70)** | **1.44 (1.40, 1.49)** | **1.97 (1.90, 2.05)** |
| ≥4 | **23.99 (21.08, 27.30)** | **1.64 (1.59, 1.69)** | **2.46 (2.37, 2.56)** |
| Recent tobacco use |  |  |  |
| No | 1 [Ref] | 1 [Ref] | 1 [Ref] |
| Yes | **3.92 (3.70, 4.16)** | **1.79 (1.76, 1.83)** | **4.01 (3.92, 4.11)** |
| Recent alcohol use |  |  |  |
| No | 1 [Ref] | 1 [Ref] | 1 [Ref] |
| Yes | **0.29 (0.27, 0.31)** | **1.31 (1.27, 1.36)** | **2.76 (2.66, 2.87)** |
| Recent other substance use |  |  |  |
| No | 1 [Ref] | 1 [Ref] | 1 [Ref] |
| Yes | **3.86 (3.56, 4.17)** | **3.29 (3.19, 3.39)** | **6.21 (6.02, 6.41)** |
| Unknown | **2.48 (2.33, 2.65)** | **1.21 (1.18, 1.23)** | **1.87 (1.82, 1.92)** |
| Recent stress levels |  |  |  |
| No stress | 1 [Ref] | 1 [Ref] | 1 [Ref] |
| Low | **0.69 (0.53, 0.90)** | 0.95 (0.85, 1.06) | **0.78 (0.69, 0.87)** |
| Moderate | **0.53 (0.41, 0.69)** | 1.06 (0.95, 1.18) | **0.81 (0.72, 0.91)** |
| High | **0.51 (0.39, 0.66)** | 1.10 (0.98, 1.22) | **0.87 (0.77, 0.97)** |
| Positive for suicide screening |  |  |  |
| No | 1 [Ref] | 1 [Ref] | 1 [Ref] |
| Yes | **1.74 (1.64, 1.84)** | **1.33 (1.30, 1.36)** | **1.64 (1.60, 1.68)** |
| Positive for loneliness screening |  |  |  |
| No | 1 [Ref] | 1 [Ref] | 1 [Ref] |
| Yes | 1.02 (0.96, 1.08) | **1.11 (1.09, 1.14)** | **1.12 (1.10, 1.15)** |
| Positive campus climate |  |  |  |
| No | 1 [Ref] | 1 [Ref] | 1 [Ref] |
| Yes | 1.04 (0.98, 1.10) | **0.95 (0.93, 0.97)** | **0.90 (0.88, 0.92)** |

**1** adjusted odds ratio via logistic regression; bolded values represent statistically significant results (*p* < 0.05), italicized values represent results on the boundary of statistical significance (0.05 ≤ *p* < 0.10) **2** confidence interval

**Supplemental Table 6.** Associations between SUD/moderate or high cannabis SSIS (≥4) /elevated problematic cannabis involvement (score ≥2) and mental health outcomes among all students

| Outcome | Diagnosed conditions | | | | | | Receipt of mental health services | |
| --- | --- | --- | --- | --- | --- | --- | --- | --- |
|  | **Anxiety** | | **Depression** | | **Other** | |  |  |
| Exposure | **n (%^1^)** | **aPR^2^ (95% CI^3^)** | **n (%^1^)** | **aPR^2^ (95% CI^3^)** | **n (%^1^)** | **aPR^2^ (95% CI^3^)** | **n (%^1^)** | **aPR^2^ (95% CI^3^)** |
| SUD |  |  |  |  |  |  |  |  |
| No SUD | 162,044 (30.97) | 1 [Ref] | 125,755 (24.05) | 1 [Ref] | 113,529 (23.28) | 1 [Ref] | 262,926 (50.22) | 1 [Ref] |
| SUD | 5,406 (74.29) | **1.08 (1.05, 1.11)** | 5,335 (73.35) | **1.06 (1.03, 1.09)** | 5,261 (73.96) | **1.21 (1.18, 1.25)** | 6,321 (86.52) | **1.03 (1.01, 1.06)** |
|  |  |  |  |  |  |  |  |  |
| Moderate/high cannabis SSIS |  |  |  |  |  |  |  |  |
| No | 42,492 (36.99) | 1 [Ref] | 34,297 (29.88) | 1 [Ref] | 30,254 (28.16) | 1 [Ref] | 70,411 (61.07) | 1 [Ref] |
| Yes | 44,654 (46.37) | **1.03 (1.01, 1.04)** | 39,408 (40.97) | **1.06 (1.04, 1.08)** | 34,274 (37.75) | **1.06 (1.04, 1.08)** | 65,419 (67.69) | **1.03 (1.02, 1.04)** |
|  |  |  |  |  |  |  |  |  |
| Elevated problematic cannabis involvement |  |  |  |  |  |  |  |  |
| No | 144,197 (29.79) | 1 [Ref] | 109,601 (22.67) | 1 [Ref] | 99,790 (22.16) | 1 [Ref] | 237,655 (48.83) | 1 [Ref] |
| Yes | 23,367 (50.94) | **1.02 (1.01, 1.04)** | 21,565 (47.07) | **1.07 (1.05, 1.09)** | 19,111 (43.81) | **1.07 (1.05, 1.09)** | 32,394 (70.21) | **1.04 (1.03, 1.06)** |

**1** row % **2** adjusted prevalence ratio via modified Poisson regression; bolded values represent statistically significant results (*p* < 0.05), italicized values represent results on the boundary of statistical significance (0.05 ≤ *p* < 0.10) **3** confidence interval
